# Adaptive rewiring: a general principle for neural network development

**DOI:** 10.3389/fnetp.2024.1410092

**Published:** 2024-10-29

**Authors:** Jia Li, Roman Bauer, Ilias Rentzeperis, Cees van Leeuwen

**Affiliations:** ^1^ Brain and Cognition, KU Leuven, Leuven, Belgium; ^2^ Cognitive Science, RPTU Kaiserslautern, Kaiserslautern, Germany; ^3^ NICE Research Group, Computer Science Research Centre, University of Surrey, Guildford, United Kingdom; ^4^ Institute of Optics, Spanish National Research Council (CSIC), Madrid, Spain

**Keywords:** structural plasticity, brain development, generative modeling, network neuroscience, spontaneous activity, network physiology

## Abstract

The nervous system, especially the human brain, is characterized by its highly complex network topology. The neurodevelopment of some of its features has been described in terms of dynamic optimization rules. We discuss the principle of adaptive rewiring, i.e., the dynamic reorganization of a network according to the intensity of internal signal communication as measured by synchronization or diffusion, and its recent generalization for applications in directed networks. These have extended the principle of adaptive rewiring from highly oversimplified networks to more neurally plausible ones. Adaptive rewiring captures all the key features of the complex brain topology: it transforms initially random or regular networks into networks with a modular small-world structure and a rich-club core. This effect is specific in the sense that it can be tailored to computational needs, robust in the sense that it does not depend on a critical regime, and flexible in the sense that parametric variation generates a range of variant network configurations. Extreme variant networks can be associated at macroscopic level with disorders such as schizophrenia, autism, and dyslexia, and suggest a relationship between dyslexia and creativity. Adaptive rewiring cooperates with network growth and interacts constructively with spatial organization principles in the formation of topographically distinct modules and structures such as ganglia and chains. At the mesoscopic level, adaptive rewiring enables the development of functional architectures, such as convergent-divergent units, and sheds light on the early development of divergence and convergence in, for example, the visual system. Finally, we discuss future prospects for the principle of adaptive rewiring.

## 1 Introduction

After more than 30 years of major efforts since the beginning of the Decade of the Brain, there is still no universally accepted understanding of how the brain works (Mullin, 2021). However, there is some basic agreement on the characteristics of brain structure and function as revealed by network neuroscience. In particular, network neuroscience has shed light on the characteristics of brain complexity. After briefly reviewing these characteristics and the tools needed to identify them, we will argue that they evolved to solve the brain’s central problem of how to organize its internal communication. We then consider how this solution can be achieved in neurodevelopment in view of a generative model based on a simple, generic Hebbian adaptation principle known as adaptive rewiring.

Network neuroscience views the nervous system as a network of interconnected units (Bassett and Sporns, 2017; Bullmore and Sporns, 2009). Depending on the perspective, units can represent brain regions or areas at the macro level, or circuits, neurons, or even smaller components such as signaling molecules and ions at the micro level (Betzel and Bassett, 2017; Kennedy et al., 2005). Connections can accordingly be considered at different scales, such as axonal fiber bundles (Toga et al., 2012), synapses (Douglas et al., 1996), genes (Ben-Tabou de-Leon and Davidson, 2007), or microtubules (Sallee and Feldman, 2021). Connections can be further distinguished into structural ones, representing anatomical links, and functional ones, representing signaling pathways, temporal correlations (Rubinov and Sporns, 2010), or causal interactions (Friston, 2011). Network neuroscience has adopted and actively pursues each of these perspectives to advance our understanding of brain structure and function. It naturally aligns with artificial neural networks, which assign to each unit a highly dynamic activation value, and to each connection an adaptive weight that can gradually change in response to the way the activity of the network unit affects the network’s output.

A major attraction of network neuroscience is that it comes with a ready-made package of analytical tools provided by graph theory (Sporns, 2018). Graph theory focuses on the topological properties of connections (edges in graph terminology) between units (nodes or vertices in graph terminology) and provides a variety of measures to characterize network topologies, such as degree (number of edges of a node), connectedness (existence of a path of edges between two nodes), path length (number of edges on a path), and clustering coefficient (the degree to which the nodes cluster together). Applying these measures to the nervous system is a central part of network neuroscience.

These measures can monitor the continuous changes in the connectivity of the nervous system throughout its life cycle. Neuronal units grow, differentiate, or die, while their connections are subject to synaptic plasticity (Citri and Malenka, 2008), the modification of connection strengths (weights) within a given architecture, and structural plasticity (Butz et al., 2009), the formation and pruning of connections (synapses, gap junctions, or ephaptic couplings). Structural plasticity directly affects the topology of the graph. This means that we must consider the nervous system as an evolving network, in which not only connection weights, but also units and connections can be added or pruned over time.

Our main focus here is to provide an overview of a perspective that has developed over the last decades to capture the dynamics of structural plasticity, known as adaptive rewiring. To this end, we review the relevant background information, the components of adaptive rewiring, and studies that have used them to generate the complex network features that characterize biological brains. In Section 2, we discuss these features and how they might emerge from neurodevelopmental processes. In Section 3, we discuss adaptive rewiring as the fundamental dynamic principle driving these processes. In particular, we will discuss its basic mechanisms for pruning and adding connections, i.e., rewiring. In Section 4, we review some of the results of modeling adaptive rewiring. Finally, in Section 5, we discuss future directions of adaptive rewiring for applications in network neuroscience and artificial neural networks.

## 2 Small worlds and other complexities

Nervous systems are typically sparsely connected networks. For example, the human brain, with approximately 86 billion neurons (Azevedo et al., 2009) has “only” 150 trillion connections (Pakkenberg et al., 2003); to be fully connected, it would need about 50 million times more. This means that the brain has an internal communication problem. Among networks types of similar sparsity, regular networks, i.e., networks with repeating stereotyped patterns like regular lattices, have on average large path lengths (number of edges in the shortest path between two nodes) and are therefore very inefficient at long-range communication. However, they do have a high degree of clustering, which means that there is a high probability that two nodes directly connected to a common node are also directly connected to each other. This means that local communication between these nodes is likely to be efficient. On the other hand, a random network with the same sparsity has a path length that is usually orders of magnitude smaller, but lacks the benefits of clustering. The nervous system in a wide range of species is generally connected in a non-random way, but requires efficient and fast neural communication between neural units, for example in situations where an organism needs to respond to a threatening visual cue.

Watts and Strogatz (1998) showed in their seminal study that there is a family of networks that is nonrandomly connected yet has small path length. By rewiring randomly just a few connections in a regular network, its average path length decreases precipitously while the average clustering coefficient remains almost the same. They called networks with both high clustering coefficient and small path length “small-world networks” and showed that the nervous system of the nematode *Caenorhabditis elegans* is a small-world network. Later studies revealed small-world structure in the mammalian brain (macaque and mouse) in terms of interareal connections (Hilgetag and Kaiser, 2004; Sporns and Zwi, 2004) and in neuroimaging of the human brain (Bassett et al., 2006; Salvador et al., 2005; Vaessen et al., 2010).

The way in which Watts and Strogatz (1998) rewired a regular network to produce a small-world (by randomly reconnecting a subset of the connections) was not intended as a biologically plausible model for how brain networks are generated. Moreover, small-world networks are a large and diverse family, and therefore additional metrics are needed for a more meaningful characterization. In addition to being small-world, brain networks are modular (Sporns and Betzel, 2016) with a rich-club core (van den Heuvel and Sporns, 2011). A modular network consists of different pools of units (modules), with connectivity within each pool being dense and between pools being sparse. Different modules are typically connected through hubs, or high-degree nodes, and these hubs preferentially connect to each other, forming the rich club.

### 2.1 Neurodevelopmental principles could explain the complex topology of the brain

The complex network topology of a modular small world with a rich-club core can be found in the brain at different scales (e.g., Bota et al., 2015; Hamadjida et al., 2016; van den Heuvel and Sporns, 2011; Shih et al., 2015), suggesting that they are generated from a common set of principles. From here on, we refer to complex topologies of this kind as “brain-like” and reflect on what kind of principles could have led to such topologies.

Generally speaking, evolutionary selection pressures have been translated in terms of economy and efficiency. For example, Olshausen and Fields (1996) showed that a generative learning model designed to encode natural images with as few neural units as possible active at any given time eventually produces oriented receptive fields that resemble those of V1. The efficiency constraint on neural unit activity is crucial to this development. Without it, the model does not produce those receptive fields. At the beginning of the 20th century, Ramón y Cajal (1899) proposed three principal constraints in the construction of a biological nervous system: minimization of the space occupied by the neural tissue, minimization of the material, and minimization of the time of communication between different neural units or regions (Ramón y Cajal, 1899). These three principles could conflict with each other. For example, the minimization of space and material tends to produce local connections while the minimization of time favors long-range connections. The latter increases the speed and robustness of communication between distant neural elements (Bullmore and Sporns, 2012; Kaiser and Hilgetag, 2006). From this perspective, small-worlds offer a suitable trade-off, as local signaling efficiency benefits from the clustering and global signaling efficiency benefits from the short path lengths (Latora and Marchiori, 2001).

Other principles beyond those proposed by Ramón y Cajal, (1899) may additionally favor modularity and rich-club structures. As evidenced by functional and anatomical studies, the brain appears to decompose complex problems into more manageable subproblems, each of which is processed by different brain regions (Bertolero et al., 2015; Hagmann et al., 2008). These modules facilitate parallel communication, as long as hubs in the network form robust connections with each other (Meunier et al., 2010). Preferential connections between hubs forms rich-clubs that allow rapid switching of information flow between modules (Griffa and van den Heuvel, 2018). Other features that facilitate neural communication include network motifs that promote efficient neural computation (Battaglia et al., 2012; Koyama and Pujala, 2018; Sporns and Kötter, 2004) as well as convergence of signals to neural hub units and divergence from neural hubs (e.g., Jeanne and Wilson, 2015; Keller et al., 2020; Négyessy et al., 2008).

Evolutionary optimization has been a basic assumption in generations of models (e.g., Marr, 1970). But, unlike artificial systems, evolution did not design the brain like an engineer from a preconceived functional specification. As François Jacob (1977) puts it, natural selection is more like “a tinkerer who does not know exactly what he is going to produce but uses whatever he finds around him whether it be pieces of string, fragments of wood, or old cardboards; in short it works like a tinkerer who uses everything at his disposal to produce some kind of workable object.” The nervous system is full of suboptimal tinkering solutions. For example, in the human retina, retinal ganglion cells, the output hubs of the retina, are positioned on top of the layer of photoreceptor cells, so that their outgoing nerve bundle partially occludes the photoreceptor cells, creating a blind spot on the retina. For evolution, the mantram is: “good enough to survive.” Genetic variation has limited material and already established structure to work with, and operates on a hit or miss basis with no optimality criterion in sight. Sometimes these conditions might eventually provide optimal solutions, sometimes not. So, we should not jump to the conclusion that the ubiquity of complex structure in the nervous system is the product of a meticulous optimization plan. However, we might assume that it provides mechanisms to improve on what it finds.

While the evolutionary history of larger and more complex brains, such as those of mammals, as embedded in their genetic code plays a role in shaping the connectivity structure of their nervous system, it cannot instruct neuron by neuron how to assemble the entire structure, as this is beyond the capacity of the genome (Zador, 2019). Instead, the genome merely sets the stage and provides the general rules for neurodevelopmental processes that actively unfold the complex connectivity structure of the brain network with the environment also playing a vital role in shaping it (Hiesinger and Hassan, 2018). As a schematic example, the brain would need one rule, e.g., each neuron connects to its four neighbors, to form the regular network in Watts and Strogatz (1998), and the environment can further shape it into a small-world network by randomly rewiring its connections with a small probability.

The process by which a network would gradually improve its efficiency can be described in models of the generative type (Betzel and Bassett, 2017). Generative models often take the form of an algorithm that prescribes how to achieve a structure in incremental steps. Such algorithms could provide versatile heuristics for empirical hypotheses about how such structures could be produced within physical constraints and those set by development and evolution (Rubinov, 2023).

Many of these models focus on the growth of neuronal connectivity (Akarca et al., 2021; Betzel et al., 2016; Liu et al., 2024). For example, a generative network model of axonal growth, i.e., the expansion and initial attachment of synapses, using a principle of dynamic axon expansion based on attractive guidance cues produces some hallmarks of brain-like architecture such as modular small-worlds (but no rich club), in addition to lognormal distribution of connection strengths and fiber bundling (Liu et al., 2024). Tunable parameters allow for individual and/or regional variation, where gene expression combined with systematic environmental variation and stochastic fluctuations tune the parameters.

Axonal growth is a crucial first step in the development of neural network complexity. However, it is unclear how it can provide a system with the characteristic rich-club core that is present before birth (Ball et al., 2014). Nor does it provide network motifs such as convergence of inputs to a source or divergence of outputs from a source (e.g., Jeanne and Wilson, 2015; Keller et al., 2020; Négyessy et al., 2008). It appears that the growth principles, as we currently understand them, are not sufficient to produce the functional circuits required for efficient computation.

In addition to axonal growth, another important principle of neural development is activity-dependent refinement (Pan and Monje, 2020). Network structure has traditionally been assumed to be shaped by learning, which encodes neural activity patterns induced by external inputs into brain connectivity (e.g., Hubel and Wiesel, 1970; Sengpiel et al., 1999). However, several functional neural circuits are established even before birth (Kirkby et al., 2013). During early development, cortical areas generate structured spontaneous activity in the absence of sensory stimulation or motor behavior (Wu et al., 2024; Yuste et al., 2024). The specific patterns of spontaneous activity play an instructive role in the development of neural circuits (Kirkby et al., 2013; Matsumoto et al., 2024).

We propose that throughout the nervous system, spontaneous activity is the driving force that leads to its complex network structure. The synergy between neural activity and network structure bootstraps the formation of complex structural connectivity: while network structure constrains neural activity, neural activity helps to improve network structure for communication (Rubinov et al., 2009a). Spontaneous activity of the nervous system is an often-overlooked factor in generative models. As in the generative models of connectivity growth, the emergence of brain complexity via spontaneous activity-driven network restructuring is a matter of self-organization, where genetic expression, along with systematic and random and environmental factors control the parameters of spontaneous activity and structural plasticity.

In brain development, we encounter specificity, robustness and flexibility. The principle by which spontaneous activity guides development must be specific, in the sense that the system can be tailored for specific computational needs, and robust, in the sense that it allows network complexity to emerge dynamically from a range of genetic parameters and be resilient in the face of perturbations (Hiesinger and Hassan, 2018). We will, at least for the time being, classify sensory input as one of the perturbations, as the emergent brain-like architecture persists through learning. On the other hand, the principle must also be flexible, i.e., capable of generating different network topologies activated by different genetic parameters, and allow random variability of architectures across brain areas and across individuals (Bauer et al., 2021).

In the remainder of this review, we document our contribution to the development of generative models of brain functional architecture. More than 20 years ago, a series of modeling studies were initiated (Gong and van Leeuwen, 2003; Gong and van Leeuwen, 2004; van den Berg and van Leeuwen, 2004) that drew attention to the principle of adaptive rewiring as a key mechanism of how spontaneous activity shapes the brain network architecture. Adaptive rewiring is a general rule of structural plasticity in the spirit of the Hebbian principle: “What fires together wires together.” These and subsequent studies have shown how successive adaptive rewiring of initially random networks generates complex, brain-like structures. Such structures can be tuned to specific needs, emerge robustly under perturbations, and parametric or random variation leads to a variety of architectures. Adaptive rewiring thus satisfies the criteria of specificity, robustness and flexibility, and may serve as a principle for the nervous system to dynamically evolve into an efficient information propagation and integration system.

## 3 Adaptive rewiring

**FIGURE 1:**
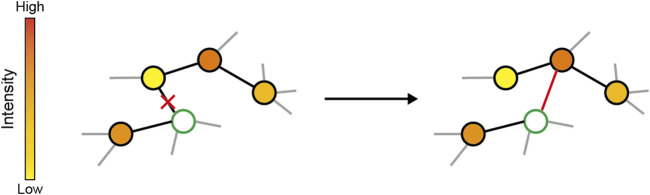
Schema of adaptive rewiring. In each step, a random node (green) is selected and its communication intensity with other nodes are computed, represented by the colors of nodes. The connection to the least interactive node is cut and added to the highest interactive but indirectly linked one.

The principle of adaptive rewiring is simple, and reminiscent of the Hebbian principle: it adds connections between nodes with high, but indirect, interaction and cuts connections between nodes with low interaction (Figure 1). The type of interaction depends on how the dynamics of activity in the network are modeled. One approach is to consider the oscillatory nature of neuronal activity, i.e., up/down states for single neurons (Wilson, 2008) and the oscillatory local potential fields in neuronal populations (Schnitzler and Gross, 2005). Early studies of adaptive rewiring models used logistic maps as nodes to mimic oscillatory neural activity (Box 1; Gong and van Leeuwen, 2003; Gong and van Leeuwen, 2004; Haqiqatkhah and van Leeuwen, 2022; Hellrigel et al., 2019; Rubinov et al., 2009a; van den Berg et al., 2012; van den Berg and van Leeuwen, 2004). For an isolated node, its activity can be modeled according to a logistic map, typically in the chaotic regime. A uniform coupling strength parameter reflects the extent to which the activity of a node is influenced by that of its neighbors (Ito and Kaneko, 2001). When coupled with other logistic maps, the chaotic dynamics are moderated by the net input from neighboring nodes, which acts as noise that dampens the oscillator. At the same time, as the coupled units become synchronized due to the coupling, chaos returns, pushing against the synchrony. As a result, the network enters a regime of intermittent dynamic synchrony with traveling and standing waves, interspersed with periods of irregular activity, a regime characteristic of spontaneous brain activity (Ito et al., 2005).

In this simple model, connections are undirected and unweighted. In a later study, the model was extended to include edge weights (Box 1; Hellrigel et al., 2019), which control the relative influence of a node’s neighbors on the node’s dynamics. In parallel, adaptive rewiring through synchrony has been studied in a spiking neuron model (Kwok et al., 2007). This model also has unweighted, directed connections. In both cases, synchrony between nodes is used as the measure of interaction strength (Fries, 2015; Palmigiano et al., 2017), defined as the absolute difference between node states for coupled logistic maps and the number of spike coincidences for spiking neural networks. Neuronal activity was assumed to operate on a shorter time scale than adaptive rewiring. Thus, network activity was allowed to evolve for a period of time prior to each rewiring step. We refer to the resulting adaptive rewiring scenario as synchrony-based adaptive rewiring.

**Box 1 Network of logistic maps.**

Let $x\left(t\right)$ be the node state at time t . The logistic map is

$$f\left(x\left(t\right)\right)=1-a{x\left(t\right)}^{2}$$

where a is the oscillatory amplitude that determines the asymptotic behavior of the logistic map.

In an undirected, unweighted network, the network dynamics follows

$$x_{i}\left(t+1\right)=\left(1-\epsilon \right)f\left(x_{i}\left(t\right)\right)+\frac{\epsilon }{\left|N_{i}\right|}\sum_{j\in N_{i}}f\left(x_{j}\left(t\right)\right)$$

where ϵ is the coupling strength, $N_{i}$ is the neighbors of node i , i.e., nodes directly connecting to node i , and $\left|N_{i}\right|$ is the number of node i ’s neighbors.

In an undirected, weighted network, the network dynamics follows

$$x_{i}\left(t+1\right)=\left(1-\epsilon \right)f\left(x_{i}\left(t\right)\right)+\frac{\epsilon }{\sum_{j\in N_{i}}w_{ij}}\sum_{j\in N_{i}}w_{ij}f\left(x_{j}\left(t\right)\right)$$

where $w_{ij}$ is the weight of the edge between node i and j .

The dynamics of individual nodes in coupled logistic maps and spiking neural networks are explicitly defined. Modeling traffic on a network directly avoids making specific assumptions about the dynamics and interaction strengths are represented by the flows between nodes. For undirected networks, network traffic is modeled by diffusion (Box 2; Calvo Tapia et al., 2020; Jarman et al., 2017; Rentzeperis and van Leeuwen, 2020), which assumes there is signal propagation between two connected nodes whenever there is a difference in their state, i.e., concentration (Kondor and Lafferty, 2002). This choice may be justified by studies showing that network diffusion has the highest explanatory power for the correlation between structural connectivity and brain activity, compared to other assumptions of signal propagation in networks (e.g., Abdelnour et al., 2014; Seguin et al., 2023). It allows for a closed-form specification of node states; a linear map quantifies the flow between nodes during a set elapsed time. By creating shortcut connections in regions of high traffic and pruning where traffic is low, adaptive rewiring optimizes the network flow. We refer to this way of modeling adaptive rewiring as diffusion-based.

Note that in these undirected networks, the interactions between nodes are symmetric, a simplification that does not account for the interactions between neurons with chemical synapses. A generalization that is a more realistic account of neuronal interactions uses directed networks (Li et al., 2024; Luna et al., 2024; Rentzeperis et al., 2022) and replaces diffusion with consensus (Ren et al., 2007) and advection (Chapman, 2015) as the measure of incoming and outgoing flows respectively (Box 2). Consensus dynamics naturally extends the diffusion equation by only considering incoming-links of nodes, i.e., a node will adjust its state according to its incoming neighbors’ states. In contrast, advection dynamics assumes links transporting a substance, the amount of which is measured by node states, along with their directions. In the special case when the connections between nodes are bilateral and equally weighted, consensus-advection dynamics reduces to the diffusion dynamics.

**Box 2 Models of information flows on networks.**

The diffusion process on an undirected network could be described by the following equation:

$${\dot{x}}_{i}\left(t\right)=\sum_{j\in N_{i}}w_{ij}\left(x_{j}\left(t\right)-x_{i}\left(t\right)\right)$$

Let A be the adjacency matrix of the network and $x\left(t\right)=\left(x_{1}\left(t\right),\ldots ,x_{n}\left(t\right)\right)$ be the vector of node states (concentrations) at time t . The sum of edge weights of a node i , $\sum_{j\in N_{i}}w_{ij}$ , is node i ’s strength. The graph Laplacian matrix, L , is defined as D−A , where D is a diagonal matrix with node strengths as diagonal entries. Then the matrix form of the diffusion equation is

$$\dot{x}\left(t\right)=-Lx\left(t\right)$$

with its closed-form solution:

$$x\left(t\right)=e^{-Lt}x\left(0\right)$$

The $\left(i,j\right)$ entry of the linear map $e^{-Lt}$ measures the flow between node i and j during the period of time t .

Consensus dynamics naturally extend the diffusion equation by only considering incoming-links of nodes,

$${\dot{x}}_{i}\left(t\right)=\sum_{\left\{j:j\to i\right\}}w_{ij}\left(x_{j}\left(t\right)-x_{i}\left(t\right)\right)$$

i.e., a node will adjust its state according to its incoming neighbors’ states. In contrast, advection dynamics assumes links transporting a substance, the amount of which is measured by node states, along with their directions.

$${\dot{x}}_{i}\left(t\right)=\sum_{\left\{j:j\to i\right\}}w_{ij}x_{j}\left(t\right)-\sum_{\left\{k:i\to k\right\}}w_{ki}x_{i}\left(t\right)$$

The in- and out-strength of node i is the total weight of its incoming-links and outgoing-links respectively. Let the in-Laplacian matrix $L_{in}$ be $D_{in}-A$ , where $D_{in}$ is a diagonal matrix with the in-strengths as diagonal entries, and the out-Laplacian matrix $L_{out}$ be $D_{out}-A$ , where $D_{out}$ is a diagonal matrix with the out-strengths as diagonal entries. Analogously to the undirected case, the closed-form solution of consensus dynamics is

$$x\left(t\right)=e^{-L_{in}t}x\left(0\right)$$

The closed-form solution of advection dynamics is

$$x\left(t\right)=e^{-L_{out}t}x\left(0\right)$$

We use the $\left(i,j\right)$ entry of $e^{-L_{in}t}$ to measure the incoming flow to node i from node j and the $\left(i,j\right)$ entry of $e^{-L_{out}t}$ to measure the outgoing flow from node j and i during the period of time t .

At least until now, the process of adaptive rewiring typically started from randomly connected, sparse networks. This assumption is made for simplicity and generality. It is complementary to the growth and initial connection assumptions. Regardless of the measure used for the interaction between nodes, the basic procedure of the adaptive rewiring algorithm is outlined below. At each step, a node is randomly selected and its interaction with other nodes is measured. A new edge is then established from the selected node to the node with the highest interaction strength among those not directly connected to the selected node. The edge connected to the node with the lowest interaction strength among the neighbors of the selected node is pruned. The principle also works on regular architectures (Rubinov et al., 2009b) and can transform networks that already have small-word properties reached under different parametrizations (Rentzeperis and van Leeuwen, 2021). Note that for simplicity, the number of nodes and connections is kept constant, but this is not essential. There are versions of the model where the number of nodes and connections grows (Gong and van Leeuwen, 2003) or is pruned (van den Berg et al., 2012). Similar to the study by Watts and Strogatz (Watts and Strogatz, 1998), a proportion of random rewiring steps are included (e.g., Jarman et al., 2017; Rentzeperis and van Leeuwen, 2020).

### 3.1 Relationship with self-organized criticality

Although formulated independently, adaptive rewiring is closely related to self-organized criticality (SOC). Criticality *per se* describes the behavior of an equilibrium system at a critical point, where the system undergoes a continuous phase transition between order and disorder (Muñoz, 2018). The hallmark of the critical state is the presence of long-range spatiotemporal correlations, that is, the state of one part of the system at a given location and time can affect distant parts or itself at later time, and the decay of the correlation function with distance or time at the critical point follows a power law (Goldenfeld, 2018). Consequently, probability distributions describing the responses to external perturbations are expected to be power laws (Jensen, 2021). The emergence of power laws is ubiquitous in natural phenomena—such as earthquakes (Gutenberg and Richter, 1950), star luminosity (Press, 1978), and coastline length (Mandelbrot, 1983)— and can be attributed to this underlying criticality.

Achieving such a state in equilibrium systems requires fine-tuning of the system’s control parameters near a critical point, which is unlikely to occur in nature. Bak et al. (1987) introduced SOC to explain how criticality can arise spontaneously in nonequilibrium systems, without the need for external tuning of control parameters. SOC posits that the critical state of a system acts as an attractor, meaning that the system naturally converges to this state through its intrinsic dynamics. Bak et al. (1987) proposed the sandpile model to demonstrate how the dynamics within such a system can generate power-law distributions in a natural, self-organized manner. The sandpile model is implemented on a lattice where each location represents a pile of sand with a given number of grains. Grains of sand are dropped randomly onto the lattice. When the number of grains at a site exceeds a certain threshold, the site “topples” and redistributes grains to its neighboring sites. This can cause neighboring sites to topple as well, resulting in a cascade of toppling events, known as an avalanche. As grains are continuously added and redistributed, the system naturally organizes itself into a critical state, where the distribution of avalanche sizes follows a power-law distribution.

The idea that neural networks might exhibit SOC was proposed even before empirical evidence for critical brain activity was available (Beggs and Plenz, 2003). Hopfield (1994) drew parallels between the SOC model for earthquakes, the Burridge-Knopoff model, and the dynamics of locally coupled integrate-and-fire neurons: In the Burridge-Knopoff model, small stresses build up locally until they cross a threshold, triggering an earthquake; in integrate-and-fire neurons, synaptic inputs accumulate until the membrane potential crosses a firing threshold, generating an action potential. Hopfield (1994) suggested that such neural networks might also exhibit SOC like the earthquake model. Building on this intuition, Herz and Hopfield showed that the sizes of synchronized clusters in these networks indeed follow a power-law distribution, the signature of criticality (Herz and Hopfield, 1995; Hopfield and Herz, 1995). Other critical phenomena, such as power-law distributed avalanche sizes (Chen et al., 1995; Corral et al., 1995; Eurich et al., 2002) and 1/f noise (Usher, 1995), have also been captured in neural networks.

The neural networks in these studies were fixed, in the sense that they did not change their connectivity and weights. To overcome this limitation, various activity-dependent mechanisms have been introduced to automatically bring the network into a critical state, e.g., Hebbian (Bienenstock and Lehmann, 1998) and homeostatic rules (Levina et al., 2007; Tetzlaff et al., 2010), reinforcement learning (Bak and Chialvo, 2001; Chialvo and Bak, 1999; de Arcangelis and Herrmann, 2010), and activity-dependent rewiring (Bianconi and Marsili, 2004; Bornholdt and Röhl, 2003; Bornholdt and Rohlf, 2000).

In the activity-dependent rewiring rule introduced by Bornholdt and Rohlf (2000), a random node is selected at each step, and based on its activity, either a new connection is created between it and another random node, or a random connection of it is pruned. Thus, rewiring occurs between two randomly selected nodes, with connection values determined by the nodes’ activity. This process drives the network density towards a value where the network activity dynamics become critical. In adaptive rewiring as described here, a random node is again selected at each step, but in contrast, links are pruned from the node with the lowest interaction intensity and added to the node with the highest interaction intensity. This is similar to the dynamics in extremal models of SOC, where the system evolves by initiating events at the unit with the extremal state value (Gabrielli et al., 1997; Paczuski et al., 1996). The adaptive rewiring rule may serve a similar function in driving the network toward a critical state.

Despite the similarities, adaptive rewiring is not intrinsically linked to criticality. The development of brain-like structures is dependent on network size and density. These must be above a certain threshold, otherwise adaptive rewiring does not guarantee the formation of local clusters (van den Berg et al., 2012). Crucially, however, above the threshold adaptive rewiring is effective for a wide range of sizes and connectivity densities. This is not because, as in SOC, criticality is an attractor for the system. Throughout adaptive rewiring, networks exhibit nonequilibrium dynamics. Adaptive rewiring networks can be viewed as autopoietic systems, where brain-like structures emerge and are maintained under a variety of conditions (Maturana and Varela, 1991). We can observe phase transitions in the network structure during its evolution. For example, networks that have settled into a centralized configuration may transition to a more modular one, while maintaining their brain-like connectivity structure (Rentzeperis and van Leeuwen, 2021). It is precisely because adaptive rewiring does not rely on SOC that it can produce specific, robust and flexible results (Rentzeperis and van Leeuwen, 2021).

## 4 Manifestations of brain network topologies

### 4.1 Emergence of brain-like complex structures

Applying iterative synchrony-based adaptive rewiring to either random (Gong and van Leeuwen, 2003; Gong and van Leeuwen, 2004; Kwok et al., 2007) or regular networks with lattice structures (Rubinov et al., 2009b) yields networks that are modular small-worlds (van den Berg and van Leeuwen, 2004; Rubinov et al., 2009a), with the modules communicating through a rich-club core (Hellrigel et al., 2019). Interesting, the evolution towards these complex, brain-like structures depends on network dynamics. In random networks of coupled logistic maps, the network dynamics is chaotic when the coupling strength is weak and gradually become ordered as the coupling strength increases (Manrubia and Mikhailov, 1999), where intermediate coupling strengths produce dynamics with intermittent or fuzzy synchronization. These are the conditions where the small-world structures emerge (Gong and van Leeuwen, 2004). The emerging structural connectivity patterns follow the functional connectivity of dynamic synchrony patterns in the model, but not the moment-to-moment ones. The averaged synchrony pattern over a moving temporal window is the driving force that generates the brain-like structural networks (Rubinov et al., 2009a).

The dynamic synchrony patterns that are effective in creating brain-like connectivity in the models correspond in the brain to the intermittent episodes of globally correlated activity observed in spontaneous activity (Gong et al., 2007; Ito et al., 2007). They allow the systems to spontaneously and rapidly enter and exit different synchrony states, and are a possible mechanism for the brain to flexibly switch between different cognitive states (Rodriguez et al., 1999). Similar dynamics have also been observed in cultured hippocampal neurons (Antonello et al., 2022; Penn et al., 2016). Their ubiquity suggests that evolution may have recruited them to support adaptive rewiring in the establishment of the brain’s network connectivity structure. Adaptive rewiring, in turn, supports this type of activity in these models, in the sense that as a result, the networks exhibit dynamic synchronization over an increasingly wide range of coupling strengths (Gong and van Leeuwen, 2004; Hellrigel et al., 2019). Thus, the title of Rubinov et al. (2009b) was apt: “Symbiotic relationship between brain structure and dynamics”.

These results were obtained in studies where synchrony-based adaptive rewiring was performed on binary networks. A study on weighted networks with fixed weights from different distributions (Gaussian or long-tailed) and with different coupling strength values generated brain-like structures for most parameterizations (Hellrigel et al., 2019). For networks with power-law weight distributions, adaptive rewiring fails to produce brain-like structures at weak coupling strengths, similarly to what has been observed in binary networks (Gong and van Leeuwen, 2004; Rubinov et al., 2009b) or below-threshold network densities (van den Berg et al., 2012). Couplings between nodes that are too sparse or too weak to sustain ordered spatiotemporal dynamics in networks fail to support the emergence of brain-like structures (Gong and van Leeuwen, 2004).

Around the density threshold, networks undergoing adaptive rewiring show interesting dynamics of their structure: clusters are formed but they are unstable (van den Berg et al., 2012). As a result, the system maintains a higher degree of randomness. A shift towards randomness were observed in the structural connectivity of anorexic patients (Collantoni et al., 2021) and in the functional connectivity of schizophrenic patients (Rubinov et al., 2009a). The dynamics of intermittent clustering and returns to randomness is reminiscent of the typical intermittent relapses and remissions of schizophrenic patients (Robinson et al., 1999). For this reason, the model behavior at the connectivity threshold has been linked to the dysconnectivity hypothesis of schizophrenia (Rubinov and Bullmore, 2013).

The emergence of a brain-like structure through adaptive rewiring is robust to perturbations in the dynamics of minority groups of nodes (Haqiqatkhah and van Leeuwen, 2022). Such groups were either collectively assigned a less chaotic oscillatory regime, thereby facilitating their synchronization to simulate sensory-driven perceptual grouping or coupled with higher strengths to represent a memory trace. When these manipulations persist, the minorities form relatively segregated modules within the overall connectivity structure. These results provide a first indication that adaptive rewiring may contribute to information processing functionality of the network.

### 4.2 Diffusion rate as a control parameter of adaptive rewiring

**FIGURE 2:**
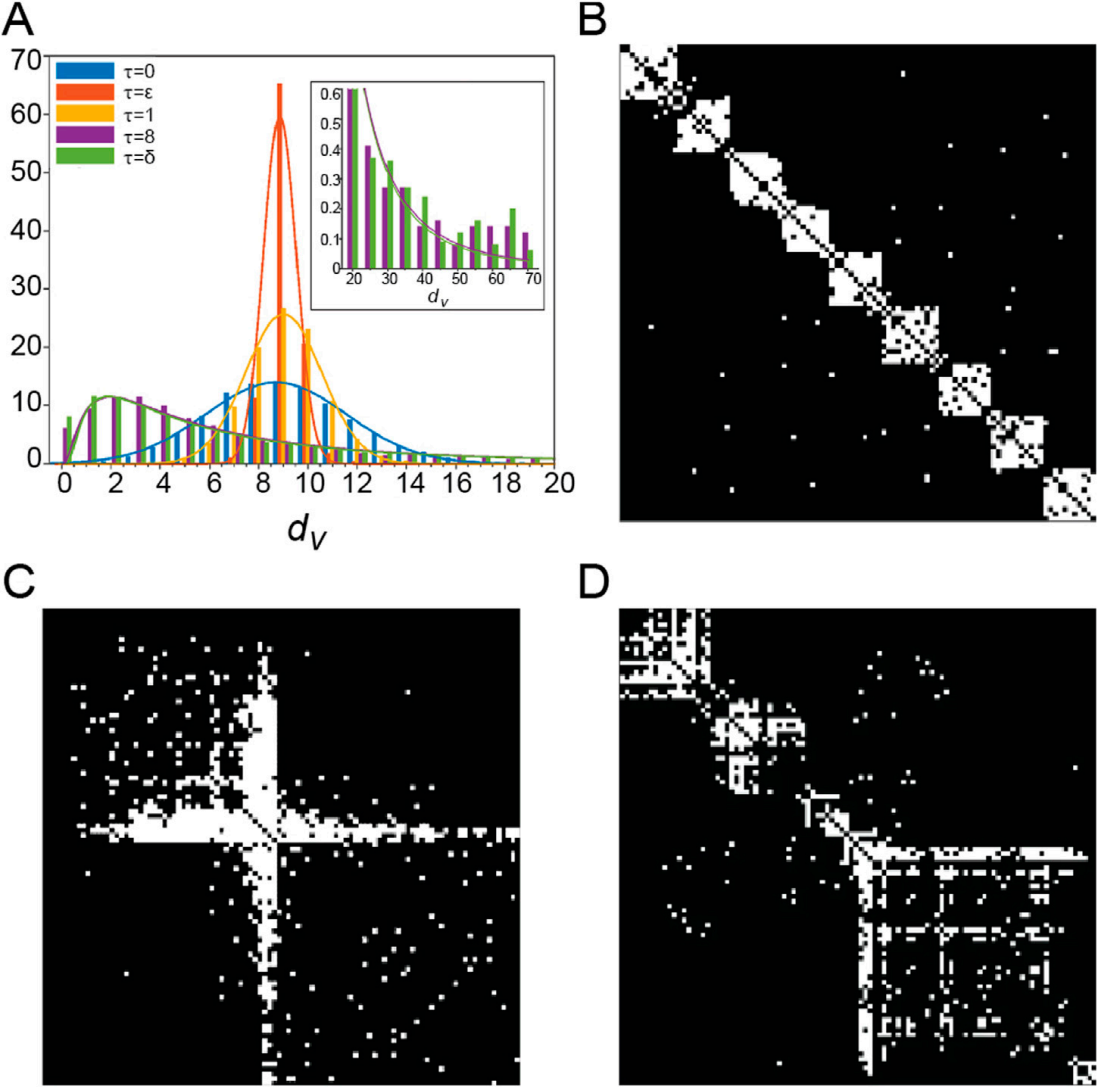
**(A)** Bar-plot of node degrees $d_{v}$ at different diffusion rate values. The height of each bar is the average number of nodes having degree $d_{v}$ . τ is the value of diffusion rate, where $\epsilon =10^{-15}$ and $\delta =10^{15}$ . For each τ , the proportion of random rewiring corresponding to the maximum the small-worldness index is chosen. **(B–D)** Examples of small-world networks with **(B)** modular, **(C)** centralized and **(D)** intermediate architectures. Adapted from Figures 1D, 2B, D, 3D in Jarman et al. (2017) under a CC BY 4.0 license.

Adaptive rewiring based on a more abstract type of interaction, diffusion, offers a parsimonious explanation for the diversity of brain topologies. Specifically, different topologies arise by varying a single parameter, the diffusion rate, defined as the elapsed time of diffusion in the network before a rewiring step occurs (Jarman et al., 2017; Rentzeperis and van Leeuwen, 2020). When the diffusion rate is low, networks develop modular structures with homogeneous degree distribution (Figures 2A, B); when it is high, networks produce centralized structures where a few high degree nodes form a core connected to peripheral nodes (Figures 2A, C); and at intermediate diffusion rates, there is a transition zone where the rewired networks exhibit some degree of both centrality and modularity (Figure 2D; Jarman et al., 2017). In all cases the rewired networks were small-worlds. This result also extends to weighted networks (normally or lognormally distributed), which also generate topological rich clubs (Rentzeperis and van Leeuwen, 2020).

Plasticity in the brain exhibits different modes, either shaping network topology to meet new computational demands or maintaining the network topology under perturbations. The former is referred to as specificity, the latter as robustness. To model different modes of brain plasticity, Rentzeperis and van Leeuwen (2021) examined the effect of diffusion rate when networks had an established brain-like topology prior to rewiring. The initial topologies varied, ranging from more modular to more centralized networks. For adaptive rewiring with small diffusion rates, the rewired networks exhibit specificity, i.e., networks become modular regardless of their initial topologies, which may involve a phase transition in case the initial topology was centralized; for intermediate and large diffusion rates, networks exhibit robustness, i.e., networks maintain their initial topologies, i.e., remain centralized if they were centralized, or remain modular if they were modular.

A third property of plasticity is flexibility. A rewiring process exhibits flexibility if it deviates stochastically from robustness or specificity. Flexibility could benefit network reorganization by establishing some diffuse connections that can be recruited, e.g., when computational demands change. Adaptive rewiring shows greater flexibility in networks with lognormally distributed weights than in those with normally distributed weights, in either the specificity or robustness mode. Recent experimental evidence suggests that synaptic strengths follow a lognormal distribution (e.g., Ercsey-Ravasz et al., 2013; Loewenstein et al., 2011; Song et al., 2005), but the advantages of this type of distribution are still unclear. The computational study by Rentzeperis and van Leeuwen (2021) offers the working hypothesis that the lognormal strength distribution contributes to the networks’ structural flexibility.

### 4.3 Synergy with spatial rewiring principles

So far, we have only considered the topological properties of brain networks, but the brain is embedded in a three-dimensional space. Incorporating this feature into computational studies will help us understand the interplay of the wiring principles that aim to minimize space, material, and processing time (Ramón y Cajal, 1899). To study these effects, network models are embedded in a three-dimensional volume with each node being assigned to a coordinate position.

A prominent spatial organization property associated with minimizing material (wiring length) is that the probability of two neurons being connected decreases with distance (Hagmann et al., 2007; Kaiser et al., 2009). As a result, neural connections are densely interconnected locally. However, to minimize processing time, brain networks also develop a number of long-range connections (Bullmore and Sporns, 2012; Kaiser and Hilgetag, 2006). These two different connectivity patterns indicate that wiring principles could be at odds with each other and that there is a happy medium where both are satisfied to a certain extent (Bullmore and Sporns, 2012).

**FIGURE 3:**
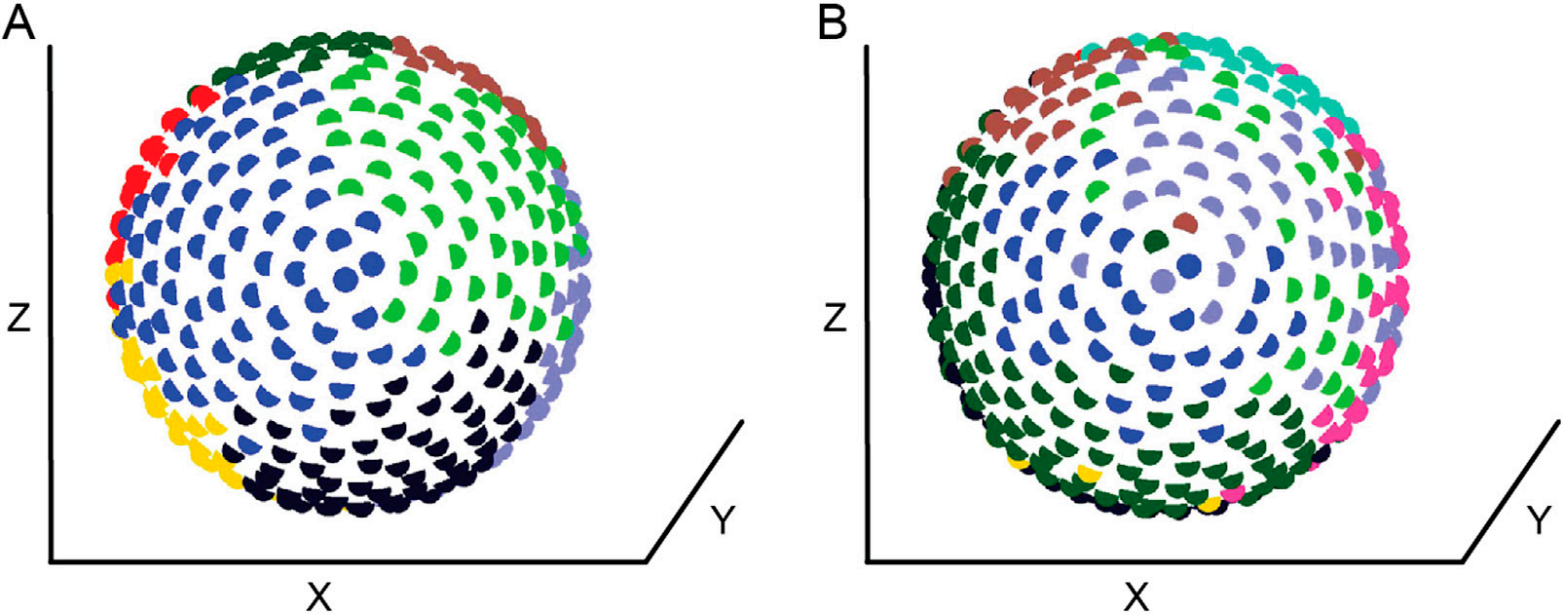
Community structure of a network after an **(A)** linear-penalizing and **(B)** under-penalizing adaptive rewiring process. Nodes are arranged in a spherical setting approximating the brain and colored according to the module to which they belong. Adapted with permission from Figures 10A, 11A in Jarman et al. (2014). Copyright 2014 by Springer Nature.

In a spatially embedded network, a rewiring penalty based on the Euclidean distance between nodes was incorporated into the synchrony-based adaptive rewiring algorithm. Complex networks evolved nevertheless, regardless of the type of cost function used (Jarman et al., 2014). Each cost function produced a degree of segregation between adjacent modules, as well as some overlap, suggesting that these structures could be involved in establishing topographical maps. However, there were some subtle differences. Adaptive rewiring based on a linear cost function yields topologically segregated modules corresponding to spatially segregated regions, consistent with observations from structural connections in the brain (Figure 3A; Hagmann et al., 2008; Rubinov et al., 2015). While the majority of connections are largely intramodular and short-range, a sparse set of long-range connections survives in the network, forming a rich-club core that connects spatially segregated modules. In contrast, an “overpenalizing” exponential cost function imposes a greater penalty on distant nodes, resulting in a stricter separation between intramodular and hub nodes. We call such networks “autistic,” based on the hypothesis that the imbalance between local and global connectivity is responsible for the precise minds characteristic of autism spectrum disorders (Belmonte et al., 2004; Van De Cruys et al., 2014). Finally, an “underpenalizing” logarithmic cost function imposes a less severe penalty on distant nodes compared to the linear and exponential cost functions. As a result, adaptive rewiring in this regime produces a less strict separation of modules and somewhat blurs the distinction between intramodular and hub nodes. Spatially, it loosens the spatial separation between modules (Figure 3B), resulting in “ectopic” nodes that are localized in the region of one module but belong to another. We named such networks “dyslexic” and “creative”: Dyslexic because the presence of ectopic nodes is likely to cause noise in modules dedicated to alphabetic reading and thus misidentification of letters; creative because the presence of ectopic units facilitates the formation of unusual conceptual combinations. The model is therefore consistent with the hypothesized relationship between creativity and dyslexia (Cancer et al., 2016; Cockcroft and Hartgill, 2004).

For diffusion-based adaptive rewiring, Calvo Tapia et al. (2020) introduced an alternative mechanism for minimizing connection length. They used a spatial proximity-based rewiring rule that replaces the longest connection of a node with a connection to the nearest unconnected node. The removal of the long-range connection is purely a consequence of the choice of using an initially random network with no biological significance. But the adherence to a neighbor may be understood as the formation of gap junctions which propagate calcium signals between spatially adjacent neurons (Niculescu and Lohmann, 2014). Rewiring occurs according to either adaptive rewiring or the spatial rewiring rule, which is determined by a Bernoulli trial at each step. A fixed proportion of spatial proximity-based rewiring maintains network connectedness while generating modular small-world structures with the modules being spatially segregated, similar to the study by Jarman et al. (2014), but this time as a function of the proportion of spatial proximity-based rewiring.

**FIGURE 4:**
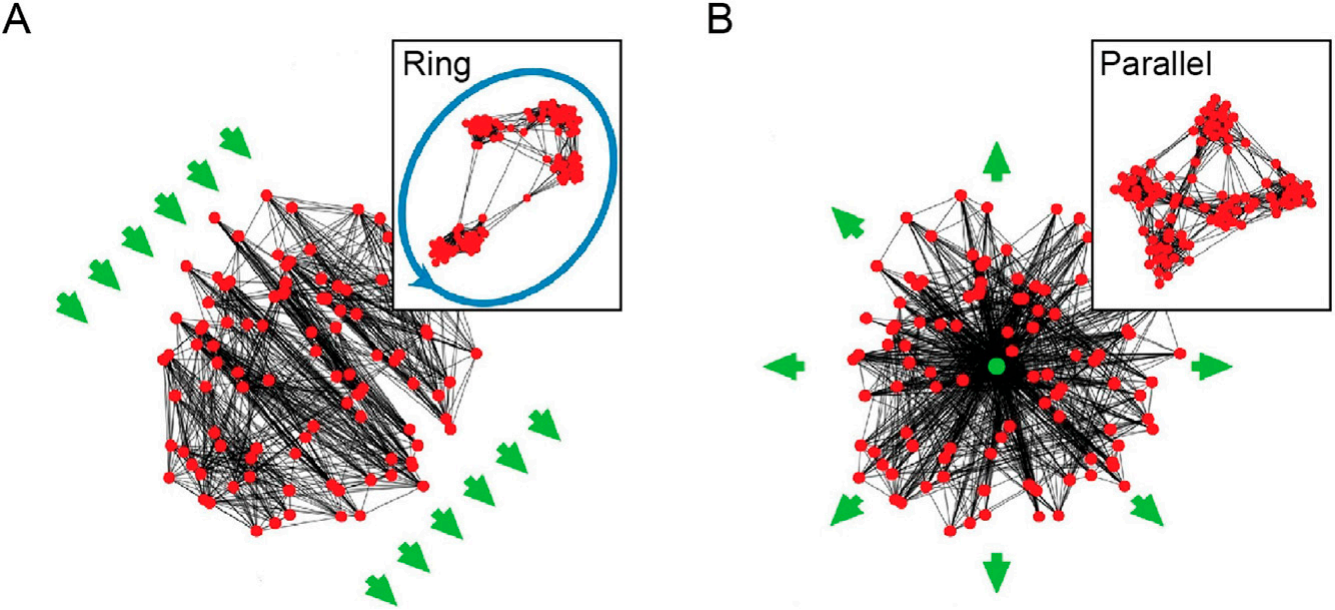
**(A)** A super-chain emerges under the influence of a laterally propagating wave. **(B)** A ganglion emerges under the influence of a radially propagating wave. Networks are embedded in a two-dimensional unit disk, where nodes are located in space according to their coordinates. Green arrows indicate the propagating waves’ direction. The insets provide a topological view where the nodes are rearranged in space to minimize the number of the crossing connections. Adapted from Figure 3 in Calvo Tapia et al. (2020) under a CC BY 4.0 license.

Another spatial organization property explored by Calvo Tapia et al. (2020) is based on the observation that connections exhibit specific non-random topographic structures. For example, neuronal connections tend to extend either in the same direction, as the axons of pyramidal cells in the cortex (Mohan et al., 2015), or extend in a concentric fashion, such as the dendrites of retinal ganglion cells (Boycott and Wässle, 1974; Völgyi et al., 2009). The formation of these topographic structures has been associated with propagating waves of electrical activity (Alexander et al., 2011; Ito et al., 2007) or gradients of guidance cues (Tessier-Lavigne and Goodman, 1996) as organizing principles. To investigate the effects of these principles, Calvo Tapia et al. (2020) introduced an alignment-based rewiring rule that aligns the connections along an underlying vector field representing propagating waves or gradients of guidance cues. When alignment-based rewiring is combined with adaptive and spatial proximity-based rewiring, the resulting network still preserves the modular small-world structure while developing a detailed brain-like functional anatomy. Namely, a laterally propagating wave organizes modules into super-chains that can serve as the structural basis for a synfire chain (Figure 4A) while a radially propagating wave organizes modules into ganglia that support parallelism with a convergent input and a divergent output (Figure 4B; McLachlan, 2003).

### 4.4 Convergent-divergent units in directed networks

Synchrony and diffusion, the measures of interaction strength introduced so far, are symmetric in that the propagation of activity flows indiscriminately in both directions of a connection. Although this symmetry is mathematically convenient and can capture certain aggregate effects, the flow of information in the nervous system is directed. Most synapses are chemical synapses, where information is transmitted unidirectionally as a neurotransmitter flows from a presynaptic to a postsynaptic terminal. In addition, structural connectivity on a larger scale (i.e., white matter tracts) is also directed (e.g., Harriger et al., 2012; Scannell et al., 1999; Varshney et al., 2011). To accommodate this anatomical asymmetry, a number of recent studies have used adaptive rewiring on directed networks (Li et al., 2024; Luna et al., 2024; Rentzeperis et al., 2022).

To model adaptive rewiring in directed networks, Rentzeperis et al. (2022) used two algorithms widely used in distributed computing: advection and consensus (Box 2; Chapman, 2015; Ren et al., 2007), both of which are generalizations of network diffusion used in undirected networks. They act as homeostatic mechanisms that aim to reduce the activity differences between units. When consensus is used to adaptively rewire the incoming links to a unit, divergent hubs are produced. Divergent hubs are units that have a large number of outgoing links and support broadcasting of information received. Similarly, when advection is used to rewire the outgoing connections to a unit, convergent hubs are generated. Convergent hubs are units that have a large number of incoming connections and support the integration of the received information.

**FIGURE 5:**
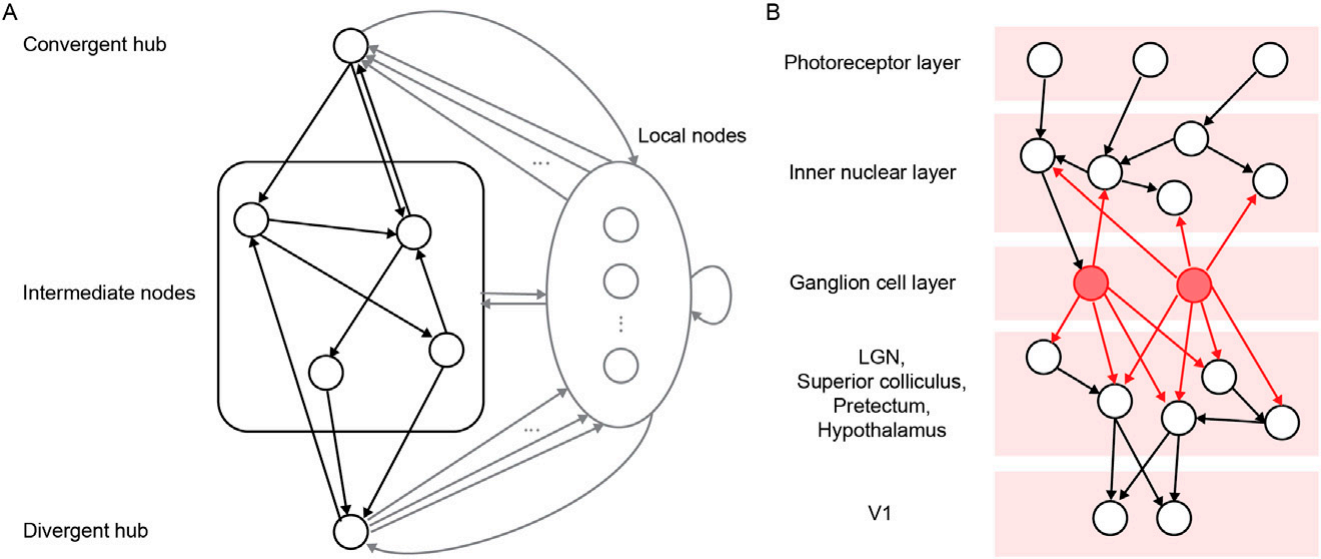
**(A)** Schema of a convergent-divergent unit. Adapted from Figure 2 in Li et al. (2024) under a CC BY 4.0 license. **(B)** Schema of retinal circuitry. Adapted from Figure 1B in Luna et al. (2024) under a CC BY 4.0 license.

When advection and consensus are used in equal proportions, the network develops convergent-divergent units (Figure 5A), consisting of convergent hubs that collect input from sparsely connected local nodes and project it through a densely interconnected, relatively encapsulated core to divergent hubs that broadcast their output back to the local units. Convergent-divergent units provide a parsimonious explanation for the emergence of context-sensitive sensory neurons, i.e., neurons that respond to local features but are also modulated by global contextual features. Prominent examples of this connectivity pattern are somatostatin (SOM) neurons which collect inputs from and project responses back to orientation-selective neurons in layers 2/3 of mouse V1. The SOM neurons and vasoactive intestinal peptide neurons form intermediate subnetworks to modulate the responses of orientation-selective neurons as the relationship between surround and stimulus changes (Keller et al., 2020). In the same study by Rentzeperis et al. (2022), a small proportion of random rewiring increased the connectivity of the directed network, in effect facilitating the communication between nodes, without affecting the convergent-divergent units. In a spatially embedded network, Li et al. (2024) showed that spatial proximity-based rewiring plays a similar role to random rewiring in promoting network connectivity. In addition, the proportion of random rewiring controlled the degree of encapsulation of the inner core of the convergent-divergent units, thus allowing them to differ in processing style.

Recently, Luna et al. (2024) used adaptive rewiring on a directed network to investigate the role of retinal waves in the formation of retinal ganglion cells (RGCs) as divergent hubs in the visual system (Figure 5B). Retinal waves are relatively structured activity dynamics that shape visual circuits (Arroyo and Feller, 2016). The authors show that such activity waves, in combination with adaptive rewiring, can generate divergent hubs, i.e., nodes that project to a large number of other nodes. These share characteristics with the connectivity profile of RGCs. In contrast, nodes that receive inputs from RGCs are disposed to become convergent hubs, which is consistent with the connectivity of the lateral geniculate nuclei (LGN) in the mammalian visual system. Indeed, RGC-targeted LGN nodes develop into convergent hubs capable of transmitting integrated signals to downstream areas, including the primary visual cortex. Thus, the model shows how retinal waves might orchestrate divergence and convergence early in the development of the visual system.

## 5 Outlook

Rather than building the brain network unit by unit, connection by connection from the genetic code, evolution has set the stage for the brain structure to unfold during development. We have discussed adaptive rewiring as a central principle in modeling how brain structure dynamically changes during development. Adaptive rewiring leads to complex brain-like networks, i.e., networks with a modular small-world structure and a rich-club core. This effect is specific in the sense that the network topology can be adapted to computational requirements, robust in the sense that the network topology is maintained under perturbations and independent of a critical regime, and flexible in the sense that it is capable of generating a variety of network configurations within the bounds of specificity and robustness. In brain development, the cost of flexibility outside of these bounds may be associated with dysfunctions such as schizophrenia, autism, and dyslexia. Adaptive rewiring was able to mimic these effects, suggesting the origin of the relationship between dyslexia and creativity. Adaptive rewiring interacts constructively with principles of spatial organization in the formation of topographically segregated modules and structures such as ganglia or chains. At the mesoscopic level, adaptive rewiring leads to the development of functional architectures, such as convergent-divergent units, and provides an explanation for the early development of divergence and convergence in the visual system.

The studies reviewed here offer a first indication of the versatility of the adaptive rewiring principle in the generative modeling of neural network structures. These models have necessarily made many simplifying assumptions to model nervous systems. Future research can extend adaptive rewiring by replacing these assumptions with more biologically plausible ones.

First, mechanisms of synaptic plasticity could be incorporated into the models. Adaptive rewiring on networks with predetermined, fixed edge weights does not take into account that synaptic strengths change continuously over time in response to specific patterns of neural activity, whether it is spontaneous or driven by external stimulation. Synaptic plasticity takes different forms that play different roles in neural development and function (Citri and Malenka, 2008). Several models have been proposed to describe how synaptic strength changes with neural activity, but they do not address the co-evolution of complex patterns of connectivity (e.g., Bi and Poo, 2001; Clopath et al., 2010; Cooper and Bear, 2012; Vogels et al., 2011). A recent generative model of synaptic self-organization, by Lynn et al. (2024) is a step in the right direction. The model uses a variation of adaptive rewiring, adaptive reweighting, to produce connectivity strengths that are heavy-tailed, matching the distributions of synaptic weights for both vertebrates and invertebrates, as well as their clustering (Lynn et al., 2024). The model iteratively prunes random connections and redistributes their strength to the remaining connections, either randomly or via a preferential growth rule. Perhaps an additional step would be to include adaptive rewiring so that the model also generates the connectivity structures discussed here.

Second, the dynamical properties of neural units, such as neuronal excitability (Picken Bahrey and Moody, 2003), of dominant frequencies of neural oscillations (Rochefort et al., 2009), and of coupling strengths (Yrjölä et al., 2024) undergo regulated changes during brain development. These temporal variations are likely involved in the transitions of spontaneous activity observed during early brain development, the precise timing of which are critical for normal development (Wu et al., 2024; Yrjölä et al., 2024). Rentzeperis and van Leeuwen (2021) showed that the effect of adaptive rewiring with different diffusion rate values on network topology can depend on the topology of initial networks. This suggests that the timing of changes in network dynamical parameters influences the results of adaptive rewiring processes. Incorporating temporal variations in network parameter values into adaptive rewiring could provide insight into how the timing of these changes affect network organization and could ultimately suggest potential targets for therapeutic intervention.

Third, adaptive rewiring could be used in combination with models of neural growth. In an early application (Gong and van Leeuwen, 2003), a generative algorithm combined a rule for random initial attachment of additional nodes with adaptive rewiring (based on oscillatory dynamics) to develop complex networks with scale-free connectivity distributions. A setting that would bring the algorithm closer to biological developmental processes would include spatial embedding and directionality, and combine adaptive rewiring with initial attachment based, for example, on dynamic axon expansion driven by guidance cues (Liu et al., 2024).

Although models of adaptive rewiring are still highly abstract, we believe that adaptive rewiring provides a suitable framework for harnessing biological principles in both neuroscience and artificial intelligence. First, adaptive rewiring could shed light on early neurodevelopment by being implemented in more biologically realistic networks. To this end, adaptive rewiring could be combined with agent-based models that capture both the biological dynamics and the physical processes of neurodevelopment, including cell proliferation, cell migration, neurite outgrowth and cell apoptosis (Bauer et al., 2014; Breitwieser et al., 2022). Given the previously highlighted role of activity-dependent processes in the construction of neural networks, adaptive rewiring can be incorporated as a mechanism of structural plasticity. Such highly biologically realistic, multi-scale computational models will provide many opportunities to validate and refine adaptive rewiring-based models with a wealth of experimental data.

In addition, adaptive rewiring could be used to study the temporal variation of functional connectivity across physiological states. Early studies using oscillatory networks have shown that networks undergoing adaptive rewiring exhibit intermittent dynamics (Gong and van Leeuwen, 2004; Hellrigel et al., 2019). These dynamics are reminiscent of the reorganizations of topology and connection strengths observed in the functional connectivity of brain rhythms (Bartsch et al., 2015; Ivanov et al., 2017; Lin et al., 2020; Liu et al., 2015). Such reorganizations are crucial for facilitating spontaneous transitions (Bartsch et al., 2015; Ivanov et al., 2017; Lin et al., 2020; Liu et al., 2015; Rosenblum, 2024) and maintaining critical temporal organization (Lo et al., 2002; Lombardi et al., 2020; Wang et al., 2019) across physiological states. As we discussed in Section 3.1, adaptive rewiring is closely related with SOC models. Therefore, adaptive rewiring may serve as a mechanism of the co-evolution of network organization and dynamics to explain these phenomena.

Finally, adaptive rewiring could be applied to deep neural networks (DNNs) as a neural architecture search method (Zoph et al., 2018; Zoph and Le, 2017). Recent advances of generative DNNs, such as ChatGPT and Sora (OpenAI, 2023; OpenAI, 2024), are impressive in their performance, but despite their remarkable achievements, even state-of-the-art DNNs still lag behind humans in many cognitive tasks (Goertzel, 2023; Maus et al., 2023; Ortega et al., 2021). Moreover, the energy consumption of these large models (Luccioni et al., 2023; Xu et al., 2024) is in stark contrast to the energy efficiency of the human brain (Balasubramanian, 2021). In particular, current DNNs typically lack the aforementioned complex network structures (Roberts et al., 2019), and differ significantly from brain networks in their neural representations (Xu and Vaziri-Pashkam, 2021). Recent studies have shown that connectome-inspired networks are comparable to or even outperform traditional DNNs in some vision tasks (Barabási et al., 2023; Bardozzo et al., 2023; Roberts et al., 2019). In contrast to state-of-the-art approaches where structural properties are determined *a priori*, adaptive rewiring allows the complexity of the network to increase with the presence of sensory inputs (Haqiqatkhah and van Leeuwen, 2022). We expect that DNNs will benefit from the brain-like network architectures generated by adaptive rewiring by more closely mimicking the efficiency of the human brain.
